# Un-reduction in field theory

**DOI:** 10.1007/s11005-017-1000-9

**Published:** 2017-09-19

**Authors:** Alexis Arnaudon, Marco Castrillón López, Darryl D. Holm

**Affiliations:** 1 Department of Mathematics, Imperial College, London, SW7 2AZ UK; 2 ICMAT(CSIC-UAM-UC3M-UCM), Facultad de Ciencias Matematicas, Deptartamento de Geometria y Topologia, Universidad Complutense de Madrid, 28040 Madrid, Spain

**Keywords:** Lagrange–Poincaré reduction, Classical field theory, Curve matching, Sigma models, 58E30, 53C05, 37J15

## Abstract

The un-reduction procedure introduced previously in the context of classical mechanics is extended to covariant field theory. The new covariant un-reduction procedure is applied to the problem of shape matching of images which depend on more than one independent variable (for instance, time and an additional labelling parameter). Other possibilities are also explored: nonlinear $\sigma$-models and the hyperbolic flows of curves.

## Introduction

Symmetry (i.e. invariance under a Lie group action) greatly facilitates the study of variational problems, both for the construction of explicit solutions of the variational equations and for their qualitative analysis. A rich variety of information arises from Lie symmetries of variational problems, especially when they are formulated geometrically. For example, a vast literature exists on the topic of reduction by symmetry. In the theory of reduction by symmetry, the idea is to take advantage of the group of symmetry to reduce the dimension of the phase space for the variational problem, thereby making the problem easier to solve. When performing such a reduction, one must provide a method for the reconstruction of the solutions of the original, un-reduced variational problem from solutions of the reduced problem. In the context of field theory, this step requires additional compatibility conditions, not needed in classical mechanics.

Surprisingly, there are instances where this procedure is more useful backwards. For example, a variational problem can appear to be rather complicated but may be recognised as the reduction by a certain group of symmetries of a simpler variational problem. Although the dimension of the corresponding un-reduced configuration space will be larger, the equations or the space itself may be simpler. Furthermore, the existence of this group of symmetries will shed light on the nature of the original equations. The idea of enlarging the configuration space of a variational problem can be found in classic references such as [23]. However, geometric mechanics enhances our understanding of reduction, by showing how the structure of the variational equations changes under regular reduction by symmetries. For example, in the theory of Lagrange–Poincaré reduction (i.e. when the configuration space is a manifold *Q* on which a Lie symmetry group *G* acts properly, see [8–10, 17]), the reduced variational equations split in two. The first equation is an Euler–Lagrange operator coupled with a gyroscopic term (the curvature of a chosen connection $\mathcal {A}$ in the bundle $Q\rightarrow Q/G$). The second equation is a conservation law, or Euler–Poincaré equation. In order to have a free variational problem in the reduced space, or equivalently Euler–Lagrange equations, one needs to introduce forces into the un-reduced principle so that the equations will decouple. The choice of this force can be made by splitting the Lagrangian into horizontal and vertical parts with respect to the connection $\mathcal {A}$. This is the un-reduction construction given in [6] for variational problems in mechanics and generalised in this article to a covariant field theoretical setting. We will also explore the interesting situations which arise when the parameter manifold is no longer simply connected.

**Fig. 1:**
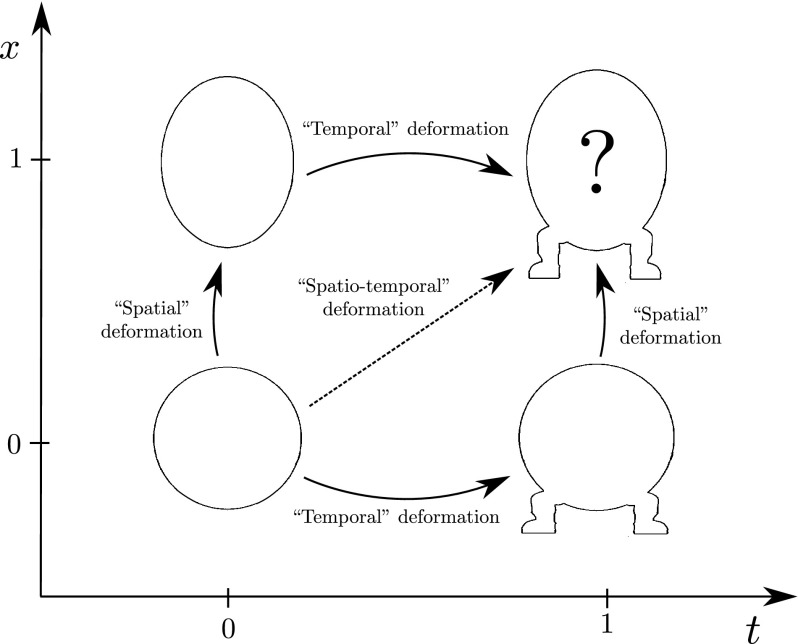
This diagram illustrates the spatio-temporal deformation of curves in $\mathrm {Sh}(S^1,\mathbb {R}^2)$ that is considered in this work. The combination of spatial and temporal deformations, where the precise meaning of space and time has to be defined depending on the context, allows for a simultaneous deformation of a curve along two parameters. The solution is then a function of (*x*, *t*) which minimises an given energy functional. In the simplest case of quadratic energy functional, the solution is known as being a harmonic map

The main motivation of [6] was in shape analysis: given two planar shapes $S_{1},S_{2}\in \mathrm {Sh}(\mathbb {R}^{2})$, understood as closed curves in $\mathbb {R}^{2}$, they seek the optimal path of shapes joining $S_{1}$ to $S_{2}$. This problem is also analysed in [12, 29] and references therein. The shape space $\mathrm {Sh}(\mathbb {R}^{2})$ is a complicated infinite dimensional manifold but in fact $\mathrm {Sh}(\mathbb {R}^{2})=Q/G$, where $G=\mathrm {Diff}^{+}(S^{1})$, and *Q* is the space $\mathrm {Emb}^{+}(S^{1},\mathbb {R}^{2})$ of positively embedded parametrisations of the circle in the plane. The space of embeddings turns out to be a simpler functional space than the shape space $\mathrm {Sh}(\mathbb {R}^{2})$. By means of conveniently chosen forces, one may use the un-reduction scheme to lift the problem of curve matching to $\mathrm {Emb}^{+}(S^{1},\mathbb {R}^{2})$. In simpler terms, this means that the parametrisations of the curves are now included in the configuration space. For field theories, this situation becomes richer. In particular, we can study matching of shapes depending on, say, two independent variables. A primary case is where the shapes depend on time and another parameter, for example labelling a set of subjects in a research study. This so-called spatio-temporal analysis of shapes is a recent and active field of research. We will briefly explain this method here and refer the reader to [14, 19, 31] for more details. In spatio-temporal shape analysis, there are two main approaches: the time-specific and subject-specific approaches. Their difference is in the variable which parametrises the evolution in shape comparisons; either for a certain subject at a sequence of times, or for a sequence of subjects at a certain time. The two approaches to the spatio-temporal construction are illustrated in Fig. 1, which shows that either of the two methods may be obtained from the other by exchanging the roles of the *x* and *t* variables. Here, in the context of field theory, the evolution of both *x* and *t* is studied together in a single equation. Note that the number of these variables is not limited to be 2, although we will often discuss two-dimensional examples for the sake of simplicity. A remarkable extension of the spatio-temporal matching is also found in [14] where the authors build a subject-specific approach together with a time reparametrisation, with interesting applications to the compared evolution of *Homo Sapiens Neanderthalensis *and *Homo Sapiens Sapiens*, or bonobos and apes. However, the deformations are not completely general in [14], because of certain statistical constraints. The configuration space of this approach is $\mathrm {Diff}(\mathbb {R}^2)$ together with the time reparametrisation in $\mathrm {Diff}(\mathbb {R})$.

Another improvement compared to [6] is with the use of a Riemannian metric in the space of embeddings that depends on derivatives of the curve (i.e. a Sobolev metric), which is a more appropriate metric for the dynamical evolution of curves. We refer to [3, 4] for more details on this topic. In [1], the authors further investigated this approach with a simple numerical test in the classical mechanical setting, but more work would be needed in order to obtain a reliable numerical scheme.

Apart from the motivation which arises from curve matching, we point out two other completely different areas of mathematical physics where covariant un-reduction is already present. The first example arises in the study of $\sigma$-models, introduced in [18], which consist of harmonic maps with values in homogeneous spaces *G* / *H*. Here, we will show that the $\sigma$-models may also be written as an un-reduction problem in *G*. Interestingly, we may sometimes combine the un-reduction method with another Euler–Poincaré reduction and obtain a different set of equations written on a vector space instead of a homogeneous space. These equations have already appeared in the literature. However, the understanding of their geometric meaning is enhanced, by using a concatenation of un-reduction and reduction. In a second example, covariant un-reduction is applied to hyperbolic curve evolution, regarded here as a simple geometric construction of other more sophisticated geometric flow equations.

*Plan and main contents of the paper* Section 2 reviews the basic concepts of covariant Lagrange–Poincaré reduction, before formulating the main result of the paper, which is the un-reduction theorem 2, in Sect. 3. Section 4 provides examples of explicit applications of the un-reduction theorem for curve matching in the plane in Sect. 4.1, nonlinear $\sigma$-model in Sect. 4.2 and finally hyperbolic curve evolution in Sect. 4.3. Each of these examples demonstrates the method of un-reduction and illustrates different ways to take advantage of the geometry of the reduced space.

## Covariant Lagrange–Poincaré reduction

The main result of the paper will be formulated as Theorem 2 in the next section. This section first reviews the basic concepts of covariant Lagrange–Poincaré reduction. The version of this reduction in mechanics takes place when a Lie group of symmetries *G* acts properly on the configuration manifold *Q* of the variational problem under study (for example see [10]). In the field theoretical setting, the group of symmetries acts on a fibre bundle $\pi : E \rightarrow N$ by vertical diffeomorphisms, that is, actions such that $\pi (y\cdot g)=\pi (y), \forall y\in E, g\in G$. We refer the reader to [8, 9, 17] for the exposition of the theoretical framework of this procedure. For our purposes, in this article we have adapted these results as follows. On one hand, we just consider trivial bundles $Q\times N \rightarrow N$, so that the dynamical objects of interest are mappings from *N* to *Q* and the problem is defined by a first-order Lagrangian defined in the first jet space $J^1(N,Q)$. This simplification is mainly done for convenience in the applications, although the theoretical core of this work can be done in full generality. On the other hand, we need to incorporate forces to our scheme, which will induce new terms in the equations in a straightforward manner.

### Background material

We will review here the background notions of differential geometry that we will use throughout this work and refer the reader to [20, 24] for more extensive texts on differential geometry and field theory.

Let $\pi : Q\rightarrow Q/G=\Sigma$ be a *G*-principal bundle where the action $R_g : Q \rightarrow Q$, $g\in G$, is assumed to be on the right. Recall that a principal connection $\mathcal {A}$ is a $\mathfrak {g}$-valued 1-form in *Q* such that the equivariance property $R_{g}^{*}\mathcal {A} =\mathrm {Ad}_{g^{-1}}\circ \mathcal {A}$ holds, and $\mathcal {A(\xi }_{Q})=\mathcal {\xi }$, for any $\xi \in \mathfrak {g}$, where $\xi _{Q}$ is the infinitesimal generator of the right action $R_g$, i.e. $\xi _{Q}(q):=d/d\varepsilon |_{\varepsilon =0}R_{\exp (\varepsilon \xi )}(q)$. This definition is equivalent to a choice of *G*-invariant splitting of the tangent bundle *TQ* into horizontal and vertical parts

$$\begin{aligned} T_{q}Q=H_{q}Q\oplus V_{q}Q, \end{aligned}$$

for $q\in Q$, where $V_{q}Q=\{(\xi _{Q})_{q} :\xi \in \mathfrak {g}\}$ and $H_{q}Q=\ker \mathcal {A}$. We denote by $p^{h}:TQ\rightarrow HQ$ and $p^{v}:TQ\rightarrow VQ$ the induced projections. The curvature of $\mathcal {A}$ is defined to be the $\mathfrak {g}$-valued two form $\mathcal {B}=d\mathcal {A}+[\mathcal {A},\mathcal {A}]$ and satisfies the equivariance property $(R_g)^*\mathcal {B}=\mathrm {Ad}_{g^{-1}}\circ \mathcal {B}$. One can also define a 2-form in $\Sigma$, but taking values in the adjoint bundle $\tilde{\mathfrak {g}}=(Q\times \mathfrak {g})/G$ as

$$\begin{aligned} \mathcal {\bar{B}}(u_{\rho },w_{\rho })=\left[ q,\mathcal {B}\left( u^{h}_{q},u^{h}_{q}\right) \right] _{G},\qquad u_{\rho },w_{\rho }\in T_{\rho } \Sigma , \end{aligned}$$

where $u^{h}_q$ stands for the unique tangent vector (the horizontal lift of $u_q$ with respect to $\mathcal {A}$) in $H_{q}Q$ such that $T\pi \left( u^{h}_q\right) =u_\rho$. The definition does not depend on $q\in \pi ^{-1}(\rho )$ because the curvature is also equivariant.

Let *N* be an oriented manifold endowed with a volume form $\mathbf {v}$ and consider a Lagrangian function $L:J^{1}(N,Q)\rightarrow \mathbb {R}$ defined in the 1-jet space of mappings $s:N\rightarrow Q$. As the jet space $J^1 (N,Q)$ can be naturally identified with $T^*N\otimes TQ$, we will also use this representation of this space in the following. We assume that *L* is invariant with respect to the lifted action of *G* in $J^{1}(N,Q)$, defined as

$$\begin{aligned} R^{(1)}_g \left( j^1 _x s\right) := j^1 _x(R_g \circ s), \end{aligned}$$

for $g\in G$ and any (local) mapping *s*. We can thus drop *L* to the quotient to obtain a reduced Lagrangian function

$$\begin{aligned} \ell :J^{1}(N,Q)/G\simeq T^{*}N\otimes (TQ)/G\longrightarrow \mathbb {R}. \end{aligned}$$

If we fix a principal connection $\mathcal {A}$ of the bundle $Q\rightarrow Q/G$, we have a diffeomorphism

$$\begin{aligned} \alpha : (TQ)/G&\rightarrow T\Sigma \oplus \tilde{\mathfrak {g}}\\ \alpha \left( \left[ v_{q}\right] _{G}\right)&\mapsto \left( T\pi \left( v_{q}\right) ,\left[ q,\mathcal {A}\left( v_{q}\right) \right] _G\right) , \end{aligned}$$

so that the reduced phase space decomposes as

$$\begin{aligned} \left( J^1(N,Q)\right) /G&=T^*N\otimes (TQ)/G \\&\cong T^*N\otimes (T\Sigma \oplus \widetilde{\mathfrak {g}}) \\&= J^1(N,\Sigma ) \oplus (T^*N \otimes \widetilde{ \mathfrak {g}}). \end{aligned}$$

From this decomposition, the reduced Lagrangian can be written as

$$\begin{aligned} \ell :J^{1}(N,\Sigma )\oplus (T^{*}N\otimes \tilde{\mathfrak {g}})\rightarrow \mathbb {R}. \end{aligned}$$

In the following sections, we will work with variational principles which will include a force term, that is, a map $F:J^{1}(N,Q)\rightarrow T^*Q$. Now, recall that the connection $\mathcal {A}$ splits the cotangent bundle $T^*Q = V^*Q \oplus H^*Q$ which directly gives the decomposition $F=F^{h}+F^{v}$, where $F^{h}=p^{h}\circ F$ and $F^{v}=p^{v}\circ F$ with $p^v$ and $p^h$ denoting the projections of $V^*Q$ and $H^*Q$, respectively. Notice that we will use the same notation as for the projection of the tangent bundle as no confusion can occur. If in addition *F* is *G*-equivariant with respect to the action of *G* in both the source and target spaces, we can drop $F^{h}$ and $F^{v}$ to $J^1(N,Q)/G$ as

$$\begin{aligned} f^{h}&:J^{1}(N,\Sigma )\oplus (T^*N\otimes \tilde{\mathfrak {g}})\rightarrow T^*\Sigma ,\quad \mathrm {and}\\ f^{v}&:J^{1}(N,\Sigma )\oplus (T^*N \otimes \tilde{\mathfrak {g}})\rightarrow \tilde{\mathfrak {g}}^*. \end{aligned}$$

Note that for $f^h$ we use $H^*Q/G\simeq T^* \Sigma$, and for $f^v$ we have the isomorphism $VQ/G\simeq \mathfrak {\tilde{g}}$ given by $[(\xi _{Q})_{q}]_{G}\mapsto [q,\xi ]_{G}$.

Finally, we recall the definition of the canonical momentum map for the natural lift action of *G* on $T^*Q$

$$\begin{aligned} \mathbf {J}:T^*Q\rightarrow & {} \mathfrak {g}^*\\ \langle \mathbf {J} (\alpha _q),\xi \rangle _{\mathfrak {g}\times \mathfrak {g}^*}= & {} \langle \alpha _q,\xi _Q\rangle _{TQ\times TQ^*} \end{aligned}$$

where $\alpha _q\in T^*Q$, $\xi \in \mathfrak {g}$, and $\xi _Q\in TQ$. We can extend $\mathbf {J}$ trivially in the factor *TN* to obtain

1 $$\begin{aligned} \mathbf {J}: TN\otimes T^*Q \rightarrow TN\otimes \mathfrak {g}^*. \end{aligned}$$

Furthermore, if we identify $TN \simeq \wedge ^{n-1}T^*N$, $n=\mathrm {dim}(N)$, by means of a fixed volume form $\mathbf {v}$, the map $\mathbf {J}: TN\otimes T^*Q \rightarrow TN\otimes \mathfrak {g}^*$ is the covariant momentum map of field theories, see [20][Proposition 4.4].

### Lagrange–Poincaré reduction

In the sequel, we will assume that *N* is compact or that the domain of variations of the maps $s:N\rightarrow Q$ is compactly supported. We project the variational principle for *L* defined on $J^1(N,Q)$ to its quotient $J^1(N,Q)/G$ to obtain $\ell : J^1 (N,\Sigma )\times (T^*N\otimes \tilde{\mathfrak {g}})\rightarrow \mathbb {R}$. Critical solutions of this reduced variational principle are maps $\sigma : N \rightarrow T^*N\otimes \tilde{\mathfrak {g}}$ which project to maps $\rho : N \rightarrow \Sigma = Q/G$ as $\rho = \pi _{\tilde{\mathfrak {g}}} \circ \sigma$ according to the following diagram2Here, $\pi _{\tilde{\mathfrak {g}}}: T^{*}N\otimes \tilde{\mathfrak {g}}\rightarrow \Sigma$ is the projection of the adjoint bundle forgetting the $T^*N$ factor. The free variations of the initial problem provide a family of constrained variations that define a new type of variational equations. They are called Lagrange–Poincaré equations, see [8, 17] for more details on these equation in this context of field theory. The next theorem gives the Lagrange–Poincaré reduction with forces *F* which corresponds the equations in the literature when $F=0$.

#### Theorem 1

(Covariant Lagrange–Poincaré reduction with forces) Let     $\pi : Q\rightarrow Q/G =\Sigma$ be a principal *G*-bundle, $\mathcal {A}$ a principal connection and *N* a compact manifold oriented by a volume form $\mathbf {v}$. Given a map $s:N\rightarrow Q$, let $\sigma :N\rightarrow T^{*}N \otimes \tilde{\mathfrak {g}}$ be defined as

$$\begin{aligned} \sigma (x)(\omega )= [s(x),\mathcal {A}(Ts\cdot (\omega ))]_G, \end{aligned}$$

with $\omega \in T_xN, x\in N$ and let $\rho :N\rightarrow \Sigma$, $\rho (x) = [s(x)]_G = \pi _{\tilde{\mathfrak {g}}} \circ \sigma$. By considering a *G*-invariant Lagrangian $L:J^{1}(N,Q)\rightarrow \mathbb {R}$ and a *G*-equivariant force $F:J^{1}(N,Q)\rightarrow T^*Q$ the following are equivalent.

1. *s* is a critical mapping of the Lagrange–d’Alembert constrained stationary principle with free variations $\delta s$
  3 $$\begin{aligned} \delta \int _{N}L\left( s,j^{1}s\right) \mathbf {v}+\int _{N}\left\langle F\left( s,j^{1}s\right) ,\delta s\right\rangle \mathbf {v}=0. \end{aligned}$$
2. The Euler–Lagrange form of *L* satisfies the relation
  $$\begin{aligned} \mathcal {EL}(L\mathbf {v})\left( j^2 s\right) =F. \end{aligned}$$
3. $\sigma :N\rightarrow T^{*}N\otimes$ $\tilde{\mathfrak {g}}$ is a critical mapping of the stationary principle
  $$\begin{aligned} \delta \int _{N}\ell \left( j^{1}\rho ,\sigma \right) \mathbf {v}+\int _N \left\langle f^{h}\left( j^{1}\rho ,\sigma \right) , \delta \rho \right\rangle \mathbf {v}+\int _N\left\langle f^{v}\left( j^{1}\rho ,\sigma \right) ,\eta \right\rangle \mathbf {v}=0, \end{aligned}$$
  for variations of the form $\delta \sigma =\nabla ^{\mathcal {A}}\eta -[\sigma ,\eta ]+\mathcal {\bar{B}}(\delta \rho ,T\rho )\in \tilde{\mathfrak {g}}$, where $\delta \rho \in T_\rho \Sigma$ is a free variation of $\rho$ and $\eta$ is a free section of $\tilde{\mathfrak {g}}\rightarrow \Sigma$.
4. $\sigma$ satisfies the Lagrange–Poincaré equations
  4 $$\begin{aligned} \begin{aligned} \mathcal {EL}_{\rho }(\ell \mathbf {v})=f^{h}-\left\langle \dfrac{\delta \ell }{\delta \sigma },i_{T\rho }\mathcal {\bar{B}}\right\rangle ,\\ \mathrm {div}^{\mathcal {A}}\dfrac{\delta \ell }{\delta \sigma }+\mathrm {ad}_{\sigma }^{*}\dfrac{\delta \ell }{\delta \sigma }=f^{v}, \end{aligned} \end{aligned}$$
  where $\mathcal {EL}_{\rho }(\ell \mathbf {v}):J^{2}(N,\Sigma )\rightarrow T^{*}\Sigma$ is the Euler–Lagrange form of $\ell$ with respect to the variable $\rho$ only and $\mathrm {div}^{\mathcal {A}}$ stands for the covariant divergence operator defined by the connection $\mathcal {A}$.

We will not give the proof of this theorem here but only an important remark. Given a solution of the Lagrange–Poincaré equations (4), the reconstruction of a solution of the initial variational problem requires a compatibility condition. Given the map $\sigma :N\rightarrow T^{*}N\otimes \tilde{\mathfrak {g}}$ and the induced map $\rho :N\rightarrow \Sigma$, we consider the pullback principal bundle $\rho ^{*}Q\rightarrow N$ and the pullback of the connection $\rho ^{*}\mathcal {A}$. Recall that the space of connections is an affine space modelled over the vector space of $\tilde{\mathfrak {g}}$-valued 1-forms in the base manifold. We can thus consider the new connection $\mathcal {A}^{\sigma }=\rho ^{*}\mathcal {A} +\sigma$. Then, the compatibility condition is

5 $$\begin{aligned} \mathrm {Curv}(\mathcal {A}^{\sigma })=0. \end{aligned}$$

Indeed, if this condition is satisfied and the manifold *N* is simply connected then the solutions $s:N\rightarrow Q$ are the integral leaves or sections of that connection. See [8, 9, 17] for more details.

## The covariant un-reduction scheme

We are now almost ready to describe the un-reduction scheme for field theories. As in the case of mechanics (see [6]), this construction requires the Lagrangian to be decomposed into horizontal and vertical parts with respect to the connection $\mathcal {A}$.

### Vertical and horizontal Lagrangians

We first give an expanded expression of the Euler–Lagrange form $\mathcal {EL}(L):J^2(N,Q)\rightarrow T^*Q$ for an arbitrary Lagrangian $L:J^{1}(N,Q)\rightarrow \mathbb {R}$ once a linear connection $\overline{\nabla }$ in *Q* has been fixed. For that, we consider the horizontal lift $v\mapsto \hat{v}$ from *TQ* to $T(T^*N\otimes TQ)$ with respect to $\overline{\nabla }$ (the lift is done in the *TQ* part only and is trivial in the $T^*N$ factor). Then we define $\frac{\overline{\nabla }L}{ds}: J^1 (N,Q)\rightarrow T^*Q$ as

$$\begin{aligned} \left\langle \frac{\overline{\nabla }L}{d s}\left( j^1 _x s\right) ,\delta s\right\rangle _{TQ\times T^*Q}:=\mathbf {d}L\left( j^1_xs\right) \cdot \widehat{\delta s}, \end{aligned}$$

for any $\delta s \in T_qQ,\,q=s(x)$. On the other hand, we define the vertical derivative $\frac{\partial L}{\partial j^1s} : J^1(N,Q)\rightarrow TN\otimes T^*Q$ as

$$\begin{aligned} \left\langle \frac{\partial L}{\partial j^1s}(j^1_x s),\omega \right\rangle :=\left. \frac{\hbox {d}}{\hbox {d}\epsilon }\right| _{\epsilon =0}L(j^1_x s+\epsilon \omega ), \end{aligned}$$

for any $\omega \in T_x ^*N\otimes T_qQ,\,q=s(x)$. The Euler–Lagrange form is thus

6 $$\begin{aligned} \mathcal {EL}(L)\left( j^2s\right) =\frac{\overline{\nabla }L}{ds}\left( j^1s\right) -\mathrm {div}^{\overline{\nabla },\mathbf {v}}\frac{\partial L}{\partial j^{1}s}\left( j^1s\right) , \end{aligned}$$

where $\mathrm {div}^{\overline{\nabla },\mathbf {v}}$ stands for the divergence operator defined by the volume form $\mathbf v$ and the affine connection $\overline{\nabla }$. This operator acts on $T^{*}Q$-valued vector fields in *N* (note that along the map $j^1s$, $\partial L/\partial j^{1}s$ is precisely a section of $TN\otimes s^{*}T^{*}Q\rightarrow N$) and is defined as the only operator such that

$$\begin{aligned} \mathrm {div}^{\mathbf {v}}\left\langle \mathcal {X},X\right\rangle =\left\langle \mathrm {div}^{\overline{\nabla },\mathbf {v}}\mathcal {X} ,X\right\rangle +\left\langle \mathcal {X},\overline{\nabla }X\right\rangle \end{aligned}$$

for any vector field $\mathcal {X}\in TN\otimes T^*Q$ and any section vector field *X* in *TQ*.

We now assume that the Lagrangian $L:J^{1}(N,Q)=T^{*}N\otimes TQ\rightarrow \mathbb {R}$ can be decomposed as $L=L^{h}+L^{v}$ where

$$\begin{aligned} L^{h}(\omega \otimes v)=L^{h}\left( \omega \otimes p^{h}(v)\right) \qquad \mathrm {and}\qquad L^{v} (\omega \otimes v)=L^{v}\left( \omega \otimes p^{v}(v)\right) , \end{aligned}$$

for any $\omega \otimes v \in T^{*}N \otimes TQ$, with respect to the connection $\mathcal {A}$. Furthermore, as $TQ=HQ\oplus VQ$, we have

$$\begin{aligned} L^{h}:T^{*}N\otimes HQ\rightarrow \mathbb {R}\qquad \text {and}\qquad L^{v}:T^{*}N\otimes VQ\rightarrow \mathbb {R}. \end{aligned}$$

Obviously, the *G* invariance of *L* and $\mathcal {A}$ extends to the *G*-invariance of $L^{v}$ and $L^{h}$ so that they drop to the quotient as

$$\begin{aligned} \ell ^{h}:J^{1}(N,\Sigma )=T^{*}N\otimes T\Sigma \rightarrow \mathbb {R}\qquad \text {and}\qquad \ell ^{v}:T^{*}N\otimes \tilde{\mathfrak {g}}\rightarrow \mathbb {R}, \end{aligned}$$

and form the reduce Lagrangian $\ell (j^1 \rho , \sigma ) =\ell ^{h}(j^1\rho )+\ell ^{v}(\rho , \sigma )$. The variational derivatives of the complete reduced Lagrangian $\ell$ then simplify as

$$\begin{aligned} \frac{\delta \ell }{\delta j^1 \rho } = \frac{\delta \ell ^h}{\delta j^1 \rho }\qquad \mathrm {and}\qquad \frac{\delta \ell }{\delta \sigma }=\frac{\delta \ell ^v}{\delta \sigma }. \end{aligned}$$

We then consider that the linear connection $\overline{\nabla }$ in *Q* is invariant under the action of *G* so that it projects to a linear connection $\nabla$ in $\Sigma =Q/G$ by the condition $\nabla _X Y = \pi _*\big (\overline{\nabla }_{X^h} Y^h\big )$. In addition, the connection $\mathcal {A}$ induces a connection in the associated bundle $\tilde{\mathfrak {g}}\rightarrow \Sigma$. With respect to these connections, we can compute

$$\begin{aligned} \frac{\nabla \ell }{d \rho }=\frac{\nabla \ell ^h}{d \rho }+\frac{\nabla \ell ^v}{d \rho }, \end{aligned}$$

and the Lagrange–Poincaré equations (4) thus read

7 $$\begin{aligned} \begin{aligned} \mathrm {div}^{\nabla , \mathbf {v}}\left( \dfrac{\delta \ell ^h}{\delta j^1 \rho }\right) -\dfrac{\nabla \ell ^h}{\delta \rho }&= f^h + \dfrac{\nabla \ell ^v}{\delta \rho } - \left\langle \dfrac{\delta \ell ^v}{\delta \sigma },i_{T\rho }\bar{\mathcal {B}}\right\rangle \\ \mathrm {div}^{\mathcal {A}}\dfrac{\delta \ell ^v}{\delta \sigma }+\mathrm {ad}_{\sigma }^{*}\dfrac{\delta \ell ^v}{\delta \sigma }&=f^{v}. \end{aligned} \end{aligned}$$

The Lagrangian splitting is crucial in this method and allows the appearance of the standard Euler–Lagrange equations for $\ell ^h$ in the left-hand side of the first equation. The second important ingredient is the force term $f^h$ which will allow us to exactly obtain the Euler–Lagrange equations by cancelling the right-hand side of the same equation.

### The un-reduction theorem

Using the previous splitting of the Lagrangian and the corresponding Lagrange–Poincaré equations (7), we can state and prove the theorem of un-reduction in the field theory.

#### Theorem 2

(Un-reduction theorem) Let *N* be a smooth oriented manifold endowed with a volume form $\mathbf {v}$ and let $\pi : Q \rightarrow \Sigma$ be a *G*-principal bundle equipped with a principal connection $\mathcal {A}$. Let $l:J^1(N,\Sigma )=T^*N\otimes T\Sigma \rightarrow \mathbb {R}$ be a first-order Lagrangian. We consider a *G*-invariant Lagrangian $L:J^1(N,Q)=T^*N\otimes TQ\rightarrow \mathbb {R}$ such that $L=L^h + L^v$ where $L^h\circ p^h = L^h$ is uniquely determined by *l* and $L^v \circ p^v = L^v$ is arbitrary. Recall that $p^h$ and $p^v$ are the projectors of the splitting $TQ=HQ\oplus VQ$ induced by the connection $\mathcal {A}$. We also consider a *G*-equivariant force $F:J^1(N,Q)\rightarrow T^*Q$ such that $F^v=p^v \circ F$ is arbitrary and $F^h = p^h \circ F$ is given by the condition

8 $$\begin{aligned} f^h = -\frac{\nabla \ell ^v}{\delta \rho } + \left\langle \frac{\delta \ell ^v}{\delta \sigma },i_{T\rho }\bar{\mathcal {B}}\right\rangle , \end{aligned}$$

for its projection $f^h:J^1(N,\Sigma )\times (T^*N\otimes \tilde{\mathfrak {g}})\rightarrow T^*\Sigma$. Then, the variational equations of the problem defined by *L* and *F* read

9 $$\begin{aligned} \begin{aligned} \mathcal {EL}\big (L^h\big )\big (j^2 s\big )&=0\\ \mathcal {A}^* \mathrm {div}^\mathbf {v}\left( \mathbf {J}\left( \dfrac{\delta L^v}{\delta j^1 s}\right) \right)&= F^v \big (j^1 s\big ), \end{aligned} \end{aligned}$$

where $\mathcal {A}^* : \mathfrak {g}^*\rightarrow V^*Q$ is the dual of the connection. Then, the critical solutions $s:N\rightarrow Q$ of (9) project to critical solutions $\rho = [s]_G$ of the Euler–Lagrange equations $\mathcal {EL}(l)(j^2 \rho )=0$.

#### Proof

We follow the notations of the preceding sections. The variational principle of *L* and *F* is

$$\begin{aligned} 0&= \delta \int _N L^h\mathbf {v} + \delta \int _N L^v\mathbf {v} + \int _N \langle F^h ,\delta s\rangle \mathbf {v} + \int _N \langle F^v ,\delta s\rangle \mathbf {v}\\&= \delta \int _N L^h \mathbf {v} + \int _N \left\langle \frac{\delta \ell ^v}{\delta \sigma },\delta \sigma \right\rangle \mathbf {v} + \int _N \left\langle \frac{\nabla \ell ^v}{\delta \rho },\delta \rho \right\rangle \mathbf {v}\\&\quad + \int _N \left\langle f^h , \delta \rho \right\rangle \mathbf {v} + \int _N \langle F^v ,\mathcal {A}( \delta s)\rangle \mathbf {v}\\&= \delta \int _N L^h (j^1 s) \mathbf {v} + \int _N \left\langle \frac{\delta \ell ^v}{\delta \sigma }, \delta \sigma \right\rangle \mathbf {v} + \int _N \left\langle \frac{\delta \ell ^v}{\delta \sigma }, \bar{\mathcal {B}}(T\rho , \delta \rho )\right\rangle \mathbf {v}\\&\quad +\int _N \langle F^v , \mathcal {A}(\delta s)\rangle \mathbf {v}. \end{aligned}$$

From the expression of $\delta \sigma$ in Theorem 1 with $\eta (x) = (s(x),\mathcal {A}(\delta s))_G$, we have that

$$\begin{aligned} \int _N \left\langle \frac{\delta \ell ^v }{\delta \sigma },\delta \sigma + \bar{\mathcal {B}}(T\rho , \delta \rho )\right\rangle \mathbf {v}= \int _N \left\langle \frac{\delta \ell ^v }{\delta \sigma }, \nabla ^\mathcal {A}\eta - [\sigma ,\eta ]\right\rangle \mathbf {v}. \end{aligned}$$

For any $f: N\rightarrow \mathfrak {g}$, recall that the covariant derivative is $\nabla ^\mathcal {A}(s(x),f(x))_G=(s(x),df(x) + [\mathcal {A}(j^1s),f])_G=(s(x),df(x))_G +[\sigma , (s(x),f(x))_G]$. Now, for $f=\mathcal {A}(\delta s)$, we have

$$\begin{aligned}&\int _N \left\langle \frac{\delta \ell ^v }{\delta \sigma },\delta \sigma + \bar{\mathcal {B}}(T\rho , \delta \rho )\right\rangle \mathbf {v} = \int _N \left\langle \frac{\delta \ell ^v }{\delta \sigma }, (s,d\mathcal {A}(\delta s))_G\right\rangle \mathbf {v} \\&= \int _N \left\langle \mathbf {J}\left( \frac{\delta L^v}{\delta j^1 s}\right) ,d\mathcal {A}(\delta s)\right\rangle \mathbf {v} = -\int _N \left\langle \mathrm {div}^{\mathbf {v}}\left( \mathbf {J} \left( \frac{\delta L^v}{\delta j^1 s}\right) \right) ,\mathcal {A}(\delta s)\right\rangle \mathbf {v}. \end{aligned}$$

Using that $L^h(j^1 s)=l (j^1 \rho )$, the variation of the action defined by $L^h$ with respect to vertical variations of $L^h$ vanishes. The variational principle naturally splits into vertical and horizontal part to yield Eq. (9). Finally, the solutions of the variational problem defined by *M* project to solutions of the problem defined by $l=\ell ^h$ by Theorem 1. $\square$

#### Remark 3

(Reduction to classical mechanics) In the case of classical mechanics where $N=\mathbb {R}$ and $\mathbf {v}=dt$ we have $\mathrm {div}^\mathbf {v}=d/dt$ and we recover the un-reduction equations of [6].

In the un-reduction theorem, it was simpler to use the reduced expression of the horizontal force $F^h$ for the condition (8) and the corresponding form for the force $F^h$ is

10 $$\begin{aligned} F^h=-\frac{\overline{\nabla } L^v}{ds}+\left\langle \mathbf {J}\left( \frac{\partial L^v}{\partial j^1s}\right) ,i_{Ts}\mathcal {B}\right\rangle . \end{aligned}$$

The variational principle on the un-reduced space of Eq. (3) is then defined using this particular force such that the reduced Lagrange–Poincaré equations decouple.

Let us now comment on the un-reduction equation (9). The first equation in (9) is the standard Euler–Lagrange equation for the horizontal Lagrangian $L^h$ only. For the second equation which is a conservation law there is an important comment to be made that is the position of $\mathcal {A}$ and $\mathrm {div}^{\mathbf {v}}$ cannot be exchanged in general as the authors did in [6]. In fact, the divergence of $\mathcal {A}^*\mathbf {J}\big (\delta L^v/\delta j^1s\big )$ would require an additional (linear) connection in *Q*. Moreover, as we mentioned in the definition (1) of the map $\mathbf {J}$ used in this equation, we have that $\mathbf {J}\big (\delta L^v/\delta j^1s\big )$ is a covariant momentum map, so that $\mathrm {div}^{\mathbf {v}}\mathbf {J}\big (\delta L^v/\delta j^1s\big )$ is the conservation law with respect to the group of symmetries *G*. Being a conservation law, the right-hand side corresponds to forcing terms which in this case are only the vertical forces $F^v$ that may be used to externally control the dynamic along the vertical space.

#### Remark 4

(Boundary conditions) For the sake of simplicity, we have restricted ourselves to the family of compactly supported variations. This is the standard formulation in geometric field theories. However, one may also consider more general variations, such as non-compactly supported variations or variations which do not vanish along the boundary (if the manifold *N* has a boundary). In these more general cases, new terms will appear in the formulas above, and these may be easily computed by using the multisymplectic form formula, for example. See [28] for more details.

### Reconstruction and surjectivity

Recall that the un-reduction theorem 2 says that solutions of the un-reduced problem project to solutions of the Euler–Lagrange equations defined by the Lagrangian *l*. One may ask if this projection is exhaustive, that is, if every solution of the variational equations for the reduced Lagrangian *l* is a projection of a solution for the Lagrangian *L*. The solution of this question may involve topological constraints concerning *N* (see the next Sect. 3.4), but we give here an answer assuming that *N* is simply connected or by just considering this question from a local point of view. From the Lagrange–Poincaré reduction theorem 1, the variational equations defined by *L* are equivalent to

$$\begin{aligned} \mathcal {EL}(\ell ^h)(j^2\rho )=0 \qquad \mathrm {and}\qquad \mathrm {div} ^\mathcal {A} \frac{\delta \ell ^v}{\delta \sigma } + \mathrm {ad}^*_\sigma \frac{\delta \ell ^v}{\delta \sigma } = f^v, \end{aligned}$$

that is, they contain the Euler–Lagrange equations for $l=\ell ^h$ together with an additional equation which may restrict the solution of the first. This equation is written in terms of the map $\sigma : N \rightarrow T^*N\otimes \tilde{\mathfrak {g}}$ and $\rho : N \rightarrow \Sigma$. Recall that $\sigma$ determines $\rho$ as $\rho = \pi _{\tilde{\mathfrak {g}}}\circ \sigma$ (see diagram (2)) and that the first reduced equation only involves $\rho$ and its first jet $j^1\rho$. Therefore, once we have a solution $\rho$ and $j^1\rho$, we may consider both the second reduced equation and the compatibility condition which thus become equations for map $\sigma$ seen as sections of the bundle $T^*N\otimes \rho ^* \tilde{\mathfrak {g}}\rightarrow N$. This means that we “restrict” the vertical part of our construction to the fibres which sit on the solution $\rho$. With the solution of these equations, we can perform reconstruction to get a map $s:N\rightarrow Q$ such that $\rho = [s]_G$. This discussion is summarised in the following proposition.

#### Proposition 5

In the notation of Theorem 2, if the space–time domain *N* is simply connected, then the un-reduction method is surjective. That is, each solution of the Euler–Lagrange equations for the reduced Lagrangian *l* is the projection of a solution of the un-reduced equations defined by *L* and *F*.

### Topological constraints for un-reduction

The topology of the manifold *N* may create interesting situations in the reconstruction and un-reduction frameworks. If *N* is not simply connected, the compatibility condition (5) does not ensure the existence of global integral sections and the surjectivity of the projection $s \mapsto \rho$ of the solutions involves other global considerations.

An example of this situation is the following. Consider $Q=S^3$ and $G=S^1$ so that $Q\rightarrow \Sigma = S^2$ is the Hopf fibration. Choose the mechanical connection $\mathcal {A}$ in this bundle, that is, the connection such that $H_qS^3 \perp V_q S^3$ with respect to the standard Riemannian metric in $S^3$. For the sake of simplicity, we consider $N= S^1$, that is, a problem of mechanics with cyclic solutions where, in addition, the compatibility condition (5) is satisfied automatically. We denote $\theta$ the coordinate of $S^1$ and we consider the *G*-invariant Lagrangian $L:J^1(N,S^3)\rightarrow \mathbb {R}$,

$$\begin{aligned} L\big (j^1_\theta s\big )=\frac{1}{2}\left\| \dot{s}(\theta ) \right\| ^2, \end{aligned}$$

where $\dot{s} = d s /d \theta \in T _{s(\theta )}S^3$ and the decomposition $L=L^h+L^v$ is induced by the orthogonal splitting $\dot{s}(\theta ) = \dot{s}^h(\theta ) + \dot{s}^v(\theta )$ defined by the connection $\mathcal {A}$. The adjoint bundle $\tilde{\mathfrak {g}}\rightarrow S^2$ is a trivial line bundle and the reduced phase space $J^1(N,\Sigma )\times (T^*N\otimes \tilde{\mathfrak {g}})$ becomes $TS^2 \times T^*S^1$. We can also decompose the reduced Lagrangian as $\ell = \ell ^h + \ell ^v$ with

$$\begin{aligned} \ell ^h\big ( j^1 \rho \big ) = \frac{1}{2}\left\| \dot{\rho } \right\| ^2 \qquad \mathrm {and} \qquad \ell ^v (\sigma )= \frac{1}{2} \varsigma ^2, \end{aligned}$$

where $\rho : S^1\rightarrow \Sigma =S^2$, $\dot{\rho } = d \rho /d \theta$, and $\sigma = \varsigma d\theta$, $\varsigma$ being a map $S^1\rightarrow \tilde{\mathfrak {g}}\cong \mathbb {R}$. The reduced equations can directly be computed to give

$$\begin{aligned} \nabla \dot{\rho } =0\qquad \mathrm {and}\qquad \dot{\varsigma } = f^v. \end{aligned}$$

First, the solution of the first equation is a closed geodesic in $S^2$. Then, the curve $s(\theta )$ of the un-reduced problem will be in the restriction $\rho ^* S^3$ of the Hopf fibration along $\rho$. This restriction is a torus and according to the reconstruction process seen in Sect. 2.2, the curve $s(\theta )$ must be horizontal with respect to the connection $\mathcal {A}+\varsigma d\theta$. Under these circumstances, the curve $s(\theta )$ need not be closed and in fact, the phase $\varphi \in S^1$ such that $s(2\pi )-s(0)=\varphi$ is precisely the holonomy of the connection $\mathcal {A}+\varsigma d\theta$ along the curve $\rho$. The holonomy of $\mathcal {A}$ alone is $\pi$ (indeed, the connection $\mathcal {A}$ is not flat and the holonomy is related with the Chern number of the Hopf bundle, see [24, Chapter XII]). Hence, besides the conditions $\dot{\varsigma } =f^v$ and $\varsigma (2\pi )= \varsigma (0)$ for the closeness of $c(\theta )$, $\varsigma (\theta )$ must satisfy

$$\begin{aligned} \int _0 ^{2\pi } \varsigma (\theta )\mathrm{d}\theta =-\pi , \end{aligned}$$

so that the holonomy of $\mathcal {A}$ vanishes. Only very specific functions $f^v$ can fulfil these conditions. For example, $f^v(\theta )=\mathrm {cos}(\theta )$ gives $\varsigma (\theta ) =\mathrm {sin}(\theta ) -1/2$ as a possible solution but other functions $f^v$ may not provide closed curves $c(\theta )$. Furthermore, it is important to note that the constant value of the holonomy of the connection $\mathcal {A}$ along geodesics $\rho$ is rather unusual and other choices for $\mathcal {A}$ may define a holonomy that will depend on $\rho$. In that case, the choice of $f^v$ will depend on the global curve $\rho$ and will not be a local object anymore.

In other words, there are circumstances where one cannot recover all solutions of the reduced problem from those of the un-reduced problem. It seems that the freedom in the choice of $L^v$ and, especially, $F^v$ may solve this issue but their specific expression will depend on the solution $\rho$ itself. We refer the reader to [27, 30] for the study of related approaches to this problem or [32] for a similar situation but in the context of isoholonomic problems in quantum computations. The situation for manifolds *N* of dimension greater than 1 is, of course, much more involved and will not be treated here.

## Applications

### Planar curve matching

We begin the application section with curve matching, the main motivation for this work and the original un-reduction scheme initiated in [6].

#### Geometric setting

Let $Q= \mathrm {Emb}^+(S^1,\mathbb {R}^2)$ be the manifold of positive oriented embeddings from $S^1$ to $\mathbb {R}^2$. Elements in *Q* are maps $c(\theta )\in \mathbb {R}^2$ for $\theta \in S^1$ and elements in the tangent space $T_cQ$ are pairs (*c*, *v*) with $c\in \mathrm {Emb}^+(S^1,\mathbb {R}^2)$ and $u \in C^\infty ( S^1 , \mathbb {R}^2)$ a parametrised vector field along the curve *c*. Then the tangent bundle is trivial, read

$$\begin{aligned} TQ = Q \times C^\infty (S^1 , \mathbb {R}^2), \end{aligned}$$

and we can take a trivial linear connection $\overline{\nabla }$ in *Q*. We then consider an open domain $N\subset \mathbb {R}\times \mathbb {R}$ with the Euclidean metric, coordinates (*t*, *x*) and volume form $\mathbf v=dt\wedge dx$. Elements of the jet bundle $J^1(N,Q)\simeq T^*N\otimes TQ$ are written as

11 $$\begin{aligned} j^1_{(x,t)}c = c_t(\theta )(t,x) dt + c_x(\theta )(t,x)dx, \end{aligned}$$

that is, $c_t$ and $c_x$ are the derivatives of a map $c:N\rightarrow Q$ along *t* and *x*, respectively.

The symmetry of this problem is the reparametrisation of the curve, which corresponds to the group $G= \mathrm {Diff}^+(S^1)$ of orientation preserving diffeomorphisms acting of $S^1$. Its Lie algebra $\mathfrak {g}= \mathfrak {X}(S^1)$ simply consists of vector fields on $S^1$. The group *G* acts on the right in $\mathrm {Emb}^+(S^1,\mathbb {R}^2)$ as reparametrisation of the curves *c* and the reduced space is the space of shapes in $\mathbb {R}^2$, that is

12 $$\begin{aligned} \Sigma := \frac{Q}{G} = \frac{\mathrm {Emb}^+(S^1,\mathbb {R}^2)}{\mathrm {Diff}^+(S^1)}. \end{aligned}$$

The principal bundle $Q\rightarrow \Sigma$ is endowed with a canonical principal connection $\mathcal {A}$ as follows. Given $u\in T_c Q$, we consider its tangent and normal decomposition

13 $$\begin{aligned} u(\theta )=v(\theta )\mathbf {t} (\theta ) + h(\theta ) \mathbf {n}(\theta ), \end{aligned}$$

where $(\mathbf {t} , \mathbf {n})$ is the orthonormal Frenet frame along *c* and $v(\theta ), h(\theta )$ are scalar functions along the curve. We have that $v(\theta )\mathbf{t}(\theta )$ is a vector tangent to the orbits of $G=\mathrm {Diff}^+(S^1)$, i.e. $v(\theta )\mathbf{t} (\theta )\in V_cQ$. We can thus define the horizontal part of *u* to be $h(\theta ) \mathbf{n}(\theta )$ and we have a decomposition $TQ=HQ\oplus VQ$. This is the natural choice for the fibration $\mathrm {Emb}^+ \rightarrow \mathrm {Emb}^+/\mathrm {Diff}^+(S^1)$, and the connection is not trivial (that is, the curvature does not vanish).

The definition of a convenient Riemannian metric in $Q=\mathrm {Emb}^+(S^1,\mathbb {R}^2)$ that should be invariant with respect to the action of $G=\mathrm {Diff}^+(S^1)$ is an interesting topic which has attracted the attention of many recent works. See for example [3, 4] and references therein. The natural $L^2$ metric

14 $$\begin{aligned} g(u_1,u_2)=\int _{S^1} \langle u_1(\theta ) , u_2(\theta )\rangle \mathrm{d}l, \end{aligned}$$

where $u_1,u_2 \in T_c Q$ and $dl = | c_\theta |d\theta$ being the arc-length, is not very useful as it may give a zero geodesic distance in both *Q* and *Q* / *G*, see [4] for more details. This problem can be overcome in the shape space *Q* / *G* by the curvature weighted metric

15 $$\begin{aligned} g(u_1,u_2)=\int _{S^1} \big (1+A\kappa (\theta )^2\big ) \langle u_1(\theta ) ,u_2(\theta )\rangle \mathrm{d}l, \end{aligned}$$

with $A>0$ and $\kappa$ the Frenet curvature of curve *c*. The drawback of is that this metric again yields zero geodesic distances in *Q* along the fibres of the fibration $Q\rightarrow Q/G$. A metric with a well-defined Riemannian distance in both *Q* and *Q* / *G* is obtained by adding higher-order derivatives of $u_1$ and $u_2$ in a Sobolev-type expression as

16 $$\begin{aligned} g(u_1,u_2)=\int _{S^1} \left( \langle u_1(\theta ) , u_2(\theta ) \rangle + A^2\langle D_\theta u_1(\theta ) , D_\theta u_2(\theta )\rangle \right) \mathrm{d}l, \end{aligned}$$

where $D_\theta = \frac{1}{|c_\theta |}\partial _\theta$ is the arc-length derivative, invariant under reparametrisations of the curve. For simplicity here, we will encompass these three cases (as well as some others, see [3]) in the metric

17 $$\begin{aligned} g_{\mathcal {P}}(u_1,u_2) = \int _{S^1} \langle u_1(\theta ),\mathcal {P} u_2(\theta )\mathrm{d}l \rangle \end{aligned}$$

that depends on a *G*-invariant self-adjoint pseudo-differential operator $\mathcal {P}$. In particular, this operator $\mathcal {P}$ for (15) is $\mathcal {P}=1 + A\kappa (\theta )^2$ and for (16) it reads $\mathcal {P} = 1-A^2D_\theta ^2$.

Unfortunately, the mechanical connection defined by these metrics (that is, the connection such that the horizontal and vertical distributions are orthogonal) need not coincide with the natural connection given in formula (13). This problem has recently been tackled in [5] where the authors exhibited the metrics that induce the natural connection. In particular, they showed that the mechanical connection for the metrics of the type

18 $$\begin{aligned} g(u_1,u_2)&= \int _{S^1}\left( h_1(\theta )\mathcal {P}_c h_2(\theta ) + v_1(\theta )\mathcal {P}_c v_2(\theta )\right) \mathrm{d}l,\nonumber \\&\mathrm {where}\quad \mathcal {P}_c(f)=\sum _{s=1} ^ l A_s(c) (D^s_\theta f)^2, \end{aligned}$$

for arbitrary smooth functions $A_s$ is the natural connection. In addition, the metric already presents a horizontal/vertical decomposition which fits perfectly in the un-reduction scheme.

#### Reduction and un-reduction

Elements of the shape space of plane curves $\Sigma = \mathrm {Emb}^+(S^1,\mathbb {R}^2)/\mathrm {Diff}^+(S^1)$ will be denoted by $\rho$ and the elements of the jet space $J^1(N,\Sigma )=T^*N\otimes T\Sigma$ will be expressed as

$$\begin{aligned} j^1_{(t,x)} \rho = \rho _t (t,x) dt + \rho _x (t,x) dx. \end{aligned}$$

Furthermore, elements of $T^*N\otimes \tilde{\mathfrak {g}}$ are simply

$$\begin{aligned} \sigma (t,x) = \sigma _t(t,x) dt + \sigma _x(t,x) dx, \end{aligned}$$

where $\sigma _t (t,x)$ and $\sigma _x (t,x)$ belong to the adjoint bundle $\tilde{\mathfrak {g}}\rightarrow \Sigma$ and can be understood as vector fields tangent to $\rho \in \Sigma$. We consider the $\mathrm {Diff}^+(S^1)$-invariant Lagrangian $L:J^1(N,Q)\simeq T^*N\otimes TQ \rightarrow \mathbb {R}$ with the metric (18) such that $L=L^h + L^v$ with respect to the connection $\mathcal {A}$ as

19 $$\begin{aligned} \begin{aligned} L^h\big (j^1_{(x,t)}c\big )&= \frac{1}{2} \int _{S^1}\left( h_t \mathcal {P}h_t + h_x\mathcal {P} h_x \right) \mathrm{d}l,\\ L^v \big (j^1_{(x,t)}c\big )&= \frac{1}{2} \int _{S^1}\left( v_t \mathcal {P}v_t + v_x \mathcal {P} v_x \right) \mathrm{d}l, \end{aligned} \end{aligned}$$

where

$$\begin{aligned} c_t = v_t\mathbf {t} + h_t \mathbf {n} \quad \mathrm {and}\quad c_x= v_x\mathbf {t} + h_x\mathbf {n}. \end{aligned}$$

##### Remark 6

(The connection $\mathcal {A}$) Although we did not give the explicit form of the connection $\mathcal {A}$ here, as we did not need its explicit form for the derivation of the un-reduced equations, it is important that this connection is not trivial. In particular, the holonomy of the connection is non-trivial, even for the rigid motion of a circle. Interestingly, the holonomy is maximal in this particular case.

We are now ready to compute the un-reduction equation for curve matching that we write in the next proposition for $N= \mathbb {R}^2$.

##### Proposition 7

The un-reduced equations (9) for the two-dimensional problem of planar curves defined by a kinetic Lagrangian with the metric (18) are

20 $$\begin{aligned} \begin{aligned} \partial _{x}(\mathcal {P}_ch_{x}) + \partial _{t}(\mathcal {P}_ch_{t})&= D_\theta (h_{x}\mathcal {P}_c v_{x}+h_{t}\mathcal {P}_c v_{t})-\kappa H + \frac{1}{2} \left( h_x \frac{\delta \mathcal {P}_c}{\delta c} h_x + h_t \frac{\delta \mathcal {P}_c}{\delta c} h_t \right) \\ \partial _x (\mathcal {P}_c v_x)+\partial _t(\mathcal {P}_c v_t)&=F^v, \end{aligned} \end{aligned}$$

with the decomposition

$$\begin{aligned} c_x = v_x \mathbf{t}+ h_x \mathbf{n},\qquad \qquad c_t = v_t \mathbf{t}+ h_t \mathbf{n}, \end{aligned}$$

for any choice of vertical force $F^v$ and where

21 $$\begin{aligned} H:=h_x\mathcal {P}_c h_x+h_t\mathcal {P}_ch_t. \end{aligned}$$

The term $\delta \mathcal {P}_c/\delta c$ stands for the variational derivative of $\mathcal {P}_c$ with respect to $c \in \mathrm {Emb}^+(S^1,\mathbb {R}^2)$, computed with variations of the form $\delta c= \xi \mathbf {n}$. That is,

$$\begin{aligned} \frac{\delta \mathcal {P}_c}{\delta c}(f)(\xi )=\left. \frac{d}{d\epsilon }\right| _{\epsilon =0} \mathcal {P}_{c+\epsilon \xi \mathbf {n}}(f). \end{aligned}$$

##### Proof

The Euler–Lagrange equation contains two terms, the first is readily found to be

$$\begin{aligned} \mathrm {div} \frac{\delta L^h}{\delta j^1c} = \partial _t(\mathcal {P}_ch_t) + \partial _x(\mathcal {P}_c h_x). \end{aligned}$$

Before computing the second term of the Euler–Lagrange equation, we can simplify the calculation by rewriting the Lagrangian as

$$\begin{aligned} L^h\big (c,j^1c\big )|_t&= \frac{1}{2} \int _{S^1} \langle (c_t\cdot \mathbf {n})\mathbf {n}, \mathcal {P}_c(c_t\cdot \mathbf {n})\mathbf {n}\rangle \mathrm{d}l\\&= \frac{1}{2} \int _{S^1} \left( c_t\cdot J\frac{c_\theta }{|c_\theta |}\right) \mathcal {P}_c\left( c_t\cdot J\frac{c_\theta }{|c_\theta |}\right) |c_\theta |\mathrm{d}\theta , \end{aligned}$$

for the temporal part only, denoted as $$L^h|_t $$. The spatial part $$L^h|_x $$ is of the same form and is not displayed here. Since this Lagrangian is horizontal, we only consider variations of *c* that are horizontal with respect to $$\mathcal {A}$$. These are variations of the form $$\delta c= \mathbf {n} \xi $$ for $$\xi \in C^\infty (S^1)$$. With the identities $$D_\theta \mathbf {n} = -\kappa \mathbf {t}$$, $$J\mathbf {n}= -\mathbf {t}$$ and $$J\mathbf {t}= \mathbf {n}$$, we compute

$$\begin{aligned} \frac{\delta L^h|_t}{\delta c}\cdot (\mathbf {n}\xi )&= \int _{S^1} \left( c_t\cdot J(\mathbf {n} \xi )_\theta \right) \mathcal {P}_c\left( c_t\cdot \mathbf {n}\right) \mathrm{d}\theta + \frac{1}{2} \int _{S^1}h_t\frac{\delta \mathcal {P}_c}{\delta c}(h_t)\xi \mathrm{d}l \\&= \int _{S^1} \xi _\theta \left( c_t\cdot J\mathbf {n} \right) \mathcal {P}_c \left( c_t\cdot \mathbf {n}\right) \mathrm{d}\theta + \int _{S^1} \xi \left( c_t\cdot JD_\theta \mathbf {n} \right) \mathcal {P}_c \left( c_t\cdot \mathbf {n}\right) \mathrm{d}l \\&\quad + \frac{1}{2} \int _{S^1}h_t\frac{\delta \mathcal {P}_c}{\delta c}(h_t)\xi \mathrm{d}l\\&= \int _{S^1} \xi D_\theta \left[ \left( c_t\cdot \mathbf {t}\right) \mathcal {P}_c \left( c_t\cdot \mathbf {n}\right) \right] \mathrm{d}l - \int _{S^1} \xi \kappa \left( c_t\cdot \mathbf {n} \right) \mathcal {P}_c \left( c_t\cdot \mathbf {n}\right) \mathrm{d}l \\&\quad + \frac{1}{2} \int _{S^1}h_t\frac{\delta \mathcal {P}_c}{\delta c}(h_t)\xi \mathrm{d}l. \end{aligned}$$

The derivative of the Lagrangian is thus

$$\begin{aligned} \frac{\delta L^h|_t}{\delta c}= D_\theta (h_t\mathcal {P}v_t) - \kappa h_t\mathcal {P} h_t + \frac{1}{2} h_t\frac{\delta \mathcal {P}_c}{\delta c}h_t. \end{aligned}$$

From the symmetry $t\Leftrightarrow x$, we obtain the un-reduction equations (20). $\square$

For the exact form of the variational derivative of the operator $\mathcal {P}$, we refer to [7, 26]. Even without the explicit forms of these variations, though, some interesting observations can be made on the structure of these equations. Upon comparing equations (19)–(21), one may interpret the term $\kappa H$ in (20) as a penalty term for the creation of deformations of large curvature and large energy density. The sign of this term depends on the concavity or convexity of the curve, which in turn means that this term tends to make the loop circular and opposes collapse of the curve to a point. Equation (20) also shows that the dynamics in (*x*, *t*) is governed by the coupling between $h_t$ and $v_t$ required for the shape deformation to be independent of the reparametrisation.

### Horizontal Lagrangians and $\sigma$-models

The freedom in the choice of forces and Lagrangians in Theorem 2 allows us to investigate the trivial choice $L^v=0$ and $F^v=0$. From (8), the horizontal part $F^h$ of the force automatically vanishes. This simple situation appears when the un-reduced Lagrangian *L* is just the pullback of the Lagrangian $\ell ^h = l:J^1\Sigma \rightarrow \mathbb {R}$ with respect to the projection $J^1(N,Q)\rightarrow J^1 (N,\Sigma )$, $j^1s\mapsto j^1[s]_G= j^1\rho$. A solution of the problem defined by *L* is any map $s:N\rightarrow Q$ such that $\rho = [s]_G$ is a solution for *l*. This means that there is a gauge degeneracy in the sense that, given a solution *s* and any map $g:N\rightarrow G$, the map $\bar{s}=s\cdot g$ is also a solution.

Even though these trivial choices for *F* and $L^v$ are not always convenient, there are some instances where they appear naturally. This is the case of $\sigma$-models in homogeneous spaces (see for example [13, 15, 16, 21] for more details in this topic). Let $Q=G$ be a Lie group and *H* be a closed subgroup such that the Lie algebra decomposes as $\mathfrak {g}=\mathfrak {m}\oplus \mathfrak {h}$ for a certain vector space $\mathfrak {m}$ such that $[\mathfrak {h},\mathfrak {m}]\subset \mathfrak {m}$. This corresponds to a reductive decomposition $\mathfrak {m}\oplus \mathfrak {h} = \mathfrak {g} =T_eG$ that we can right translate to every $T_gG$, thus obtaining a connection $\mathcal {A}$ for the principal bundle $G\rightarrow M$ over the homogeneous space $\Sigma = M = G/H$. If we consider the harmonic Lagrangian, or $\sigma$-model

$$\begin{aligned} l:J^1(N,M )&\rightarrow \mathbb {R} \\ j^1\rho&\mapsto \frac{1}{2}\left\| d\rho \right\| ^2 , \end{aligned}$$

where the norm is taken with respect to a pseudo-Riemannian metric in *N* and a Riemannian metric in *M*. The lift *L* of the reduced Lagrangian *l* to $J^1(N,G)$ is thus

$$\begin{aligned} L:J^1(N,G )&\rightarrow \mathbb {R}\\ j^1 g&\mapsto \frac{1}{2}\left\| p^h(dg) \right\| ^2 , \end{aligned}$$

where $p^h : TG\rightarrow HG$ is the horizontal projection defined by the connection $\mathcal {A}$, the norm is taken with respect to the metric in *N* and the lift of the metric in *M* to horizontal vectors in *M*. The un-reduction theorem 2 can apply and solutions of the force-free problem defined by *L* project to the desired harmonic maps in *M*.

In the majority of the homogeneous spaces where relevant $\sigma$-models are defined, the group *G* is endowed with a bi-invariant metric. In this case, the reductive decomposition is assumed to be $\mathfrak {m}=\mathfrak {h}^\perp$ and we have a metric in *M* by imposing the projection $\pi :G\rightarrow M$ to be an isometric submersion, that is, the metric in $T_xM$ is the same as the metric in $H_gG$ for any *g* with $\pi (g)=x$. The group *G* acts on the left on the coset space *M* by isometries. Hence, the Lagrangians *l* and *L* are both *G* invariant. This group of symmetries is too big for *M* to do reduction (in fact the orbit space is a single point), but we can perform covariant Euler–Poincaré reduction for *L*. We then get a new reduced Lagrangian

$$\begin{aligned} \bar{l}:J^1(N,G)/G=T^*N\otimes \mathfrak {g}&\rightarrow \mathbb {R}\\ \varsigma&\mapsto \frac{1}{2} \left\| \varsigma _\mathfrak {m} \right\| ^2 \end{aligned}$$

where $\varsigma = \varsigma _\mathfrak {h} + \varsigma _\mathfrak {m}$ is the splitting defined by the reductive decomposition. It is easy to see that the Euler–Poincaré equation is

$$\begin{aligned} \mathrm {div}^\mathbf {v}\varsigma _\mathfrak {m} + [\varsigma _\mathfrak {h},\varsigma _\mathfrak {m}]=0, \end{aligned}$$

which together with the suitable compatibility condition can be used to get solutions of *L* that can then be projected to $\Sigma$. This approach can for example be found in [13, 16, 22]. The advantage of this un-reduction and reduction procedure is in the fact that $\mathfrak {g}$ is a vector space, which is a simpler space than either of the manifolds *G* and *M*.

The situation can even be considered in a more general framework. Let *L* be a first-order Lagrangian on a Lie group *G* which is right invariant under the action of a subgroup $H\subset G$ and left invariant under the group *G* itself. Suppose that we are interested in the induced variational problem in the homogeneous space *G* / *H*. The un-reduction and reduction procedure will first give a variational problem in *G* and then give an equation in the Lie algebra $\mathfrak {g}$ which, in general, is simpler. See [33] for a description of a similar situation in mechanics, i.e. $N=\mathbb {R}$.

### Hyperbolic curvature flow 

The hyperbolic curvature flow of plane curves (see for example [25, 34]) is the variational equation defined by the Lagrangian $L:T\mathrm {Emb}(S^{1},\mathbb {R}^{2})\rightarrow \mathbb {R}$

22 $$\begin{aligned} L(c,c_t)=\int _{S^1}\left( \frac{1}{2}\left\| c_{t}\right\| ^{2}-1\right) \mathrm{d}l. \end{aligned}$$

Note that this is not a geodesic variational principle of the $L^2$ metric which would provide null geodesic distances in both the curve and shape spaces, but a Lagrangian involving a kinetic and a potential term. Moreover, the Lagrangian *L* can be easily split into a horizontal and vertical part with respect to the connection $\mathcal {A}(c_t)\mathcal {=(}c_{t}\cdot \mathbf{t})\mathbf{t}$ as

$$\begin{aligned} L^{h}=\int _{S^1}\left( \frac{1}{2}h^{2}-1\right) \mathrm{d}l \qquad \mathrm {and}\qquad L^{v}=\int _{S^1}\frac{1}{2}v^{2}\mathrm{d}l, \end{aligned}$$

where $c_t = h \mathbf{n} + v \mathbf{t}$. The Lagrangian *L* as well as $L^h$ and $L^v$ are $\mathrm {Diff}(S^1)$-invariant as their definition is geometric and does not depend on the parametrisation of *c*.

One of the main features and applications of the hyperbolic flow (as well as of other geometric flows of curves) is the study of the corresponding evolution of the shapes of curves. If we suppose that we just want to study this evolution in the shape space $\mathrm {Emb}(S^{1},\mathbb {R}^{2})/\mathrm {Diff}^+(S^1)$, the natural Lagrangian would be $l=\ell ^h$, the projection of $L^h$ to this quotient space. In this context, the un-reduction technique applies and we have the last result of this paper.

#### Proposition 8

The un-reduced equations for the hyperbolic curvature flow with Lagrangian (22) read

$$\begin{aligned} \partial _t h =D_\theta (vh)-\kappa \left( \frac{1}{2}h^{2}-1\right) \qquad \partial _t v =F^{v} \quad \mathrm {and} \quad c_{t} = h\mathbf{n}+v \mathbf{t}. \end{aligned}$$

In particular, if we choose $F^v=0$ and a vanishing initial tangent velocity $v(0)=0$, then $v(t)=0$ for all times and the velocity of *h* is proportional to the curvature $\kappa$.

The equations of this proposition for $F^v=0$ are the hyperbolic mean flow equations that can for example be found in [25]. The usual approach in the literature uses $\mathrm {Emb}(S^1 ,\mathbb {R}^2)$ and then restricts oneself to the normal part of the flow. The approach here is based on the shapes in $\mathrm {Emb}(S^1 \mathbb {R}^2)/\mathrm {Diff}(S^1)$ so that the trivial choice of $F^v=0$ directly gives the geometric equations.

## Conclusion and open problems

Apart from having set up a precise mathematical framework for the concept of un-reduction in classical field theory, extending the work of [6], the un-reduction applied to curve matching contains interesting open problems and possible improvements that we will briefly discuss below.

*Spatio-temporal matching* The first application of our field theoretical approach for curve matching would be for matching surfaces. Indeed, we can considering a given set of slices along a cylindrical surface (a typical example would be a bone) where *x* is understood as the parameter along the main axis of the surface. The first step would be to generate the initial and final conditions by using the un-reduction scheme for the initial value problem in order to interpolate between the curves each slice. This step is made with the classical un-reduction, and the second step uses the covariant un-reduction with a shooting method in time to find the solution of the full problem, that is, a critical point of the action functional $\int L(c,c_t,c_x) \mathrm{d}t\mathrm{d}x$. In our simple case with a quadratic Lagrangian, this solution will be a harmonic map, or a minimal surface, and would then require mathematics beyond the present discussion. This model could compute the distance between two surfaces, taking into account the fact that the interpolation between the slices in space should be imposed simultaneously with the matching in time. The resulting distance will be different than a naive model, which would compute the matching in time, slice by slice. For an illustration of matching slice by slice, we refer to the last example in [11] where a surface representing a nasal cavity is reconstructed out of a set of slices. The step done in this work corresponds to the generation of initial and final surfaces only, whereas the covariant un-reduction scheme would compute the distance between these two surfaces by taking into account the temporal deformation of the entire nasal cavity.

Another application would be for the spatio-temporal analysis, recently reviewed in [14], but from yet another viewpoint. Indeed, the matching in space done in [14] does not depend on a space parameter, but is instantaneous, namely given by a single map between the two curves. In [14] they also included a “time warp” which accounts for the change of pace of the evolution of the two models to be compared. In our case, the spatial variable comes into play on the same footing as time and may thus bring more flexibility into the comparison. Again, the theory of harmonic maps could help in understanding the properties of the solutions and it would even be possible that the concept of time warp of [14] could be recovered in this context.

*Choice of the vertical force* The freedom in the choice of the vertical force in the un-reduction equations provides additional flexibility for further studies when dealing with particular examples. Different types of forces could be considered such as a force which would optimally redistribute the parametrisation along the curve so that the number of points needed for the discretisation would be optimal. Another force could be used to match the paramerisation of the target curve in order to avoid the use of the computationally more expensive method of currents.

*Implementation* In principle, the un-reduction equations in (20) can be used to solve a matching problem with a shooting method. However, the treatment of the Sobolev operator $\mathcal {P}$ in these equations and especially its inversion raises an technical difficulty. Indeed, the operator $\mathcal {P}$ is constructed using the non-standard arc-length derivative $D_\theta$ and the use of a standard Fourier transform would be problematic as the mesh along the curve is not uniform in general. At least two alternative approaches could be useful here. Either one may assume small non-uniformities of the parametrisation, so that the usual Fourier transform would be a good approximation, as done in [1], or one could use a non-uniform discrete Fourier transform. Another possible approach would be to solve the matching problem directly, by minimising the action functional $S= \int L \mathrm{d}x +\int F \mathrm{d}x$ on the space of parametrised curves, as recently implemented in [2] for example. In the last approach, however, one needs to explicitly compute the horizontal force (10) used in the un-reduction scheme.
